# Metal-Working Fluids Exposure and a Rare Frontoethmoid Lesion

**DOI:** 10.1155/2020/3148125

**Published:** 2020-08-24

**Authors:** Shayan Shahidi, Abdul Nassimizadeh, Ann Sandison, Shahzada Ahmed

**Affiliations:** ^1^University Hospital Birmingham, Birmingham B15 2TH, UK; ^2^Guys Hospital, London SE1 9RT, UK

## Abstract

This case report describes a unique nasal mass that was difficult to diagnose clinically and histologically. The patient was a middle-aged man employed as a metalworker, and he presented with a unilateral nasal obstruction and a mass arising from the right middle meatus. After a series of investigations, he underwent right-sided sphenoethmoidectomy with excision of a nasal lesion. The surgical specimen presented a major diagnostic challenge for the pathologists and clinicians involved. A series of discussions amongst two different head and neck expert teams combined with detailed clinicopathological correlation resulted in a diagnosis of a granulomatous lesion or pseudotumour related to the ingestion of water-soluble cutting oils, or “Suds oil,” as they are more commonly called. Although occupational exposures to certain inhalants, such as wood dust and formaldehyde, are well-known risk factors for sinonasal lesions, here we present a rare association between a sinonasal lesion and another inhalant, Suds oil, that has not been previously reported in the literature.

## 1. Introduction

The paranasal sinuses and nasal cavity occupy a relatively small anatomical space. Lesions arising from these two regions are typically rare and represent a histologically diverse group of tumours [1].

A number of occupational risk factors have been associated with sinonasal lesions, including exposure to wood, nickel, and formaldehyde inhalants [2]. The present case report describes the diagnostic challenges brought by a rare frontoethmoid lesion, clinically indicative of cancer, that was associated with exposure to an unknown potential occupational hazard (Suds oils).

## 2. Case Report

A 63-year-old man presented to the clinic with a two-year history of right-sided facial discomfort, medial canthal swelling, and nasal obstruction. There was no history of epistaxis or visual deficit, but he reported pain and persistent epiphora on the right side. His past medical history included ischaemic heart disease, hypertension, and diabetes. He had been taking regular analgesia for management of right-sided frontotemporal headaches. This patient was a nonsmoker and had worked as an engineer with open machines for construction of pins for 25 years.

**Figure 1:**
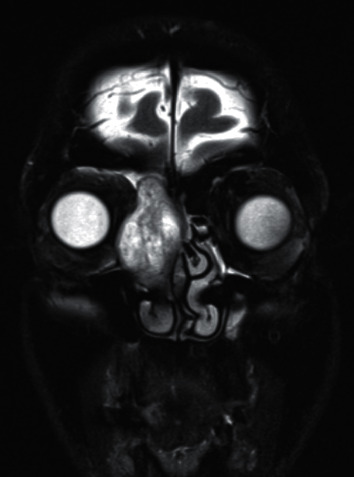
Pre-op MRI scan (coronal plane). Preoperative MRI T2 sequence showing the tumour measuring 40 × 23 mm obstructing the frontal sinus drainage pathway with infraorbital extension and bowing of the nasal septum.

On physical examination, there was a medial canthal swelling. Flexible nasal endoscopy revealed a large mass arising from the right middle meatal opening and a clear nasal cavity on the contralateral side. His magnetic resonance imaging (MRI) scans showed thinning of the medial wall of the right orbit, with evidence of a discrete mass arising from the right ethmoid sinus, extending into the right nasal cavity with likely impingement on the nasolacrimal duct (Figure 1). Subsequently, the findings were discussed in the multidisciplinary team which advised excision of the mass as opposed to an incisional biopsy because the tumour was extending into the frontal recess but seemed radiologically amenable to complete resection. Intraoperatively, a soft rubbery tumour with a discrete capsule was encountered and removed with macroscopic clearance. An initial right-sided sphenoethmoidectomy and frontal Draf IIb drill-out was performed and then extended to a Draf IIc to allow for complete removal.

**Figure 2:**
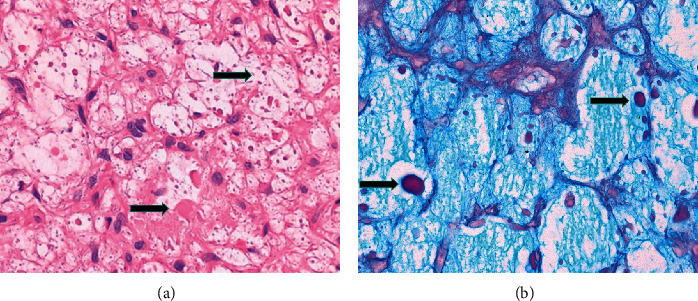
×400 hematoxylin and eosin stained section (a) and Alcian blue special stain (b). Eosinophilic material of uncertain aetiology is present in some cells.

The resected specimen was sent for histological examination which was inconclusive in the first round. The case was sent for external review to a second specialist centre for head and neck pathology in London. The second report favoured a benign process as there was no cytological atypia or marked variation in cellularity and no inflammatory cell infiltrate. The report noted numerous foamy macrophages containing eosinophilic material of uncertain aetiology. Periodic acid-Schiff (PAS)/Alcian blue special stain was negative for mucin, but the stain highlighted the eosinophilic material (Figure 2). The reporting pathologists stressed the rarity of this case and concluded that the lesion most likely represented a benign reactive granulomatous process in response to a foreign material. A detailed history revealed daily direct contact with metal-working fluids (Suds oil) and exposure to inhalation of its mist in the workplace; hence, the consultant surgeon in charge of the case suggested this oil as the most probable foreign body behind the lesion.

**Figure 3:**
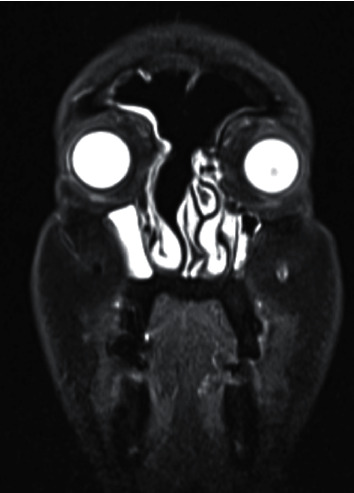
Post-op MRI scan (coronal plane). Postoperative MRI T1 with gadolinium enhancement after 6 months of follow-up. Total tumour clearance and no evidence of residuum/recurrence are confirmed.

Six months after the determination of this aetiology, a follow-up was conducted and an MRI was performed which showed no lesion residuum or recurrence (Figure 3). The patient was asymptomatic, and nasal endoscopy showed an open frontal opening with no signs of recurrence. After a 12-month follow-up, the patient was discharged from the clinic with advice to refrain from close contact with Suds oils and to check occupational health regulations regarding exposure protocols.

## 3. Discussion

Sinonasal tumours are rare, with literature reporting rates of 0.2–1% of all neoplasms and 4-5% of those arising in the head and neck regions [3, 4]. Although uncommon, they represent a histologically diverse group of tumours, with aetiology attributed to occupational, social, and genetic factors [2, 3, 5]. This histological diversity is particularly true for benign lesions [4]. This case represents a rare lesion, both in location (frontal sinus) and histology. It was clinically highly suggestive of a neoplasm and was treated as one.

The actual incidence of benign sinonasal tumours is difficult to determine, as most of these tumours are asymptomatic [4]. They can be divided into several groups, including vascular (haemangioma, juvenile angiofibroma, and pyogenic granuloma), neural-related (schwannoma, neurofibroma, and meningioma), fibro-osseous (osteoma, chondroma, ossifying fibroma, and fibrous dysplasia), hamartomatous, and inverted papilloma [4]. Osteomas, juvenile angiofibroma, and inverted papilloma are considered the most common types, with inverted papilloma accounting for 0.5–4% of nasal tumours [6]. The histological variation in sinonasal lesions often causes difficulties in sample interpretation and diagnosis, mainly because of the overlapping pathological features [3].

In this case, the histology was unusual and difficult to diagnose even for specialist head and neck pathologists in tertiary referral centres. The pathologist described a fibromyxoid proliferation and a range of differentials offered—from benign nasal glial heterotopia, chordoid meningioma, and sinonasal myxoma/fibromyxoma to low-grade malignant sarcoma and chordoma. Special histochemical stains, including Gram, PAS, and Grocott, helped exclude an infective process. A wide panel of immunostains proved nonspecific. Initially, a benign mesenchymal neoplasm with some fibroblastic/myofibroblastic differentiation was the preferred diagnosis. The supplementary report by the external experts also favoured a benign process that was not consistent with schwannoma, as suggested on imaging. The histiocytic proliferation and associated eosinophilic material led to the suggestion that the lesion represented a granulomatous reaction or pseudotumour as the most likely diagnosis among the differentials. The clinicopathological correlation was essential to support the diagnosis and for patient management.

Apart from its rare and challenging histological features, this case was associated with a potentially unrecognised occupational hazard. The association between sinonasal lesions and other occupational hazards, including exposure to wood dust and leather dust, has been widely documented [5, 7, 8]. The relative risks of sinonasal tumours have also been identified in numerous occupational settings, including the metal and machinist industries [5, 9]. Our patient developed a frontoethmoid neoplasm while working as an engineer with open machines for the construction of pins. During disease progression, he was working daily with Suds oils.

Suds oil, also called “cutting” or “straight” oil, is a petroleum-based class of metal-working fluids. They are widely used in a variety of industrial metal-working operations to improve the surface integrity of the workpiece by lubricating and reducing the friction between the tool and metal [10].

There is strong evidence that metal-working fluids (MWFs), including Suds oils, can cause skin irritation. For instance, the *International Journal of Occupational Safety* reports threefold incidence of allergic and irritant contact dermatitis in people who work closely with MWFs [11]. The other main route of exposure to MWFs is inhalation of vapour and mist, which is known to irritate the upper respiratory tract and to cause bronchitis or nasal irritation [11]. Clinical history of the patient above shows that he was exposed to Suds oil through both direct skin contact and inhalation of mist.

Considering the close daily contact with Suds oils in absence of any other historical causalities or risk factors, the consultant surgeon in charge of the case suggested this metal-working fluid as the most probable foreign body causing the granulomatous lesion described above.

We hereby present, to the best of our knowledge, the first case report of frontoethmoid pseudotumour associated with metal-working fluids. We propose a case-control study in future which measures the exposure to this potential risk factor across patients with sinonasal lesions and to examine the relationship between MWFs and the disease in more detail.

## 4. Conclusion

Sinonasal lesions are rare and histologically diverse. This case demonstrates the challenges often faced by clinicians in multidisciplinary teams managing rare sinonasal lesions. Diagnosis required numerous investigation modalities and surgical interventions and was eventually reached by consensus after careful clinicopathological correlation. This case highlights the importance of reporting challenging cases, reviewing the literature, and continuing research examining occupational hazards related to sinonasal neoplasms.

## Data Availability

No data were used to support this study.
